# Evaluating DAPI stain to assess sperm membrane integrity by flow cytometry in livestock species

**DOI:** 10.1007/s11259-025-10948-w

**Published:** 2025-10-20

**Authors:** Lydia Martínez-Rodrigo, Sabrina Gacem, Maren Brüggemann, Sandra Pinto, Eva Mocé, Daniel Serrano-Jara, M. Luz García, Jesús L. Yániz, Miguel A. Silvestre

**Affiliations:** 1 Department of Cellular Biology, Functional Biology and Physical Anthropology, University of Valencia, Burjassot, Spain; 2 Institute of Biology, University of Leipzig, Leipzig, Germany; 3 Cytometry Unit, Central Service for Experimental Research, University of Valencia, Burjassot, Spain; 4 Center of Animal Research and Technology, Valencian Institute of Agrarian Research, Segorbe, Spain; 5 CIAGRO Institute, Miguel Hernández University, Orihuela, Spain; 6 BIOFITER Research Group, Institute of Environmental Sciences (IUCA), University of Zaragoza, Huesca, Spain

**Keywords:** DAPI, Flow cytometry, Fluorochrome, Spermatozoa, Viability

## Abstract

Sperm viability is one of the key in vitro parameters of sperm quality closely related to fertility. Typically, sperm viability is assessed by flow cytometry (FC) with propidium iodide (PI) as the fluorescent stain. However, as PI emits in the red spectrum, it can overlap with other commonly used fluorochromes. Utilizing a blue viability stain, such as DAPI, for semen evaluation would free the red channel, enabling the use of additional fluorochromes and expanding analytical possibilities. This study aimed to evaluate the efficiency of DAPI as a viability stain for FC in three livestock species (goat, rabbit, and cattle), for which three experiments were conducted. First, the effect of incubation time on sperm viability rates was examined with DAPI and PI. Results indicated that incubation time did not affect the sperm permeability when DAPI and PI were employed. Second, the efficiency of DAPI as a viability indicator on different livestock species was studied. Viability rates measured with DAPI in goat, rabbit, and bull sperm samples showed significant correlation with PI measurements, showing values of 0.87, 0.86, and 0.61, respectively (*P* < 0.01). Third, the effect of intrasample variation on viability rate was analyzed. When bovine sperm samples were stained simultaneously with DAPI and PI, the correlation between the two protocols was 0.83. In conclusion, DAPI is an alternative to PI for assessing sperm viability in livestock species, whilst also highlighting the importance of possible inter-species differences.

## Introduction

Predicting the fertilizing potential of a sperm sample should constitute as the primary objective when assessing artificial insemination doses or ejaculates (Rodríguez-Martínez 2006; Silvestre et al. 2023). Among the in vitro parameters that must be evaluated in this context, sperm viability (plasma membrane integrity) is particularly critical as it correlates with field fertility outcomes (Jeyendran et al. 2019; Mendoza et al. 2021). Spermatozoa possess two distinct membranes: the acrosomal membrane and the plasma membrane. The integrity and functionality of these membranes are essential for successful fertilization as they play a central role in capacitation, oocyte binding, and the acrosomal exocytosis (Brito et al. 2003; Jeyendran et al. 2019). Loss of plasma membrane integrity renders the cell nonviable given it can no longer regulate the selective transport or permeability of essential molecules (Zubair et al. 2015).

Another technique known as “dye exclusion test” involves using stains that can be fluorescent, such as Calcein Violet or propidium iodide (PI) (Egyptien et al. 2023) or non-fluorescent, such as eosin/nigrosine or Trypan Blue (Brito et al. 2003). In this method, spermatozoa exposed to these stains are excluded if the membrane is intact. However, when membrane integrity is lost, the stains penetrate the cell and bind to their targets, labelling dead cells (Martínez-Pastor et al. 2010; Zubair et al. 2015). Among fluorescent stains, PI is one of the most used for viability assessment. PI is a non-permeable fluorochrome that binds to the DNA of dead cells, making them detectable in red fluorescent wavelength (approximately 610 nm). Typically, PI is employed alongside other permeable stains such as SYBR-14, which emits in the green spectrum and selectively binds to live cells, or Hoechst 33,342, which emits in the blue spectrum and makes it easier to differentiate cells from debris under fluorescence microscopy by binding to DNA. However, when using flow cytometry (FC), differentiating cells from debris becomes unnecessary because spermatozoa are separated based on size. This renders Hoechst or SYBR-14 unnecessary and enables the release of another channel of color emission. This allows researchers to analyze multiple parameters simultaneously using other fluorochromes (Martínez‐Pastor et al. 2010; Egyptien et al. 2023).

Nevertheless, it is important to emphasize that viability rate alone cannot fully determine the fertility potential of a seminal sample. Therefore, a compressive analysis that includes variables such as concentration, motility, acrosomal status, DNA fragmentation, mitochondrial functionality, and/or stress levels is highly recommended (Sellem et al. 2015). This approach is facilitated by adopting FC as a standard tool as it enables the simultaneous evaluation of multiple parameters (Egyptien et al. 2023). Moreover, FC has established the use of fluorescent vital staining a fundamental part of semen evaluation. This methodology not only reduces the subjectivity inherent in optical techniques but also allows one to analyse thousands of cells simultaneously (Martínez-Pastor et al. 2010). While using multiparametric FC analysis of spermatozoa offers to potentially enhance fertility prediction accuracy (De Lima Rosa et al. 2023), its application in veterinary practice remains limited probably because of the distance between the farms and the cytometry facilities. A way of implementing the use of FC on the clinical field could be by using fixable stains, which preserve labelled cells for later analysis (Peña et al. 2018). However, to successfully implement multiparametric FC analysis and ensure minimal spectral overlapping, it is crucial to identify fluorochromes emitting in blue spectrum, as most of the fluorochromes used in FC emit between the green to red spectrum (Egyptien et al. 2023).

In addition to permeable and non-permeable fluorochromes, semi-permeable fluorochromes can also be utilized if the concentration and incubation time are appropriately adjusted; this is the case of DAPI (4′,6-diamidino-2-phenylindole) (Zharkova et al. 2019) an inexpensive fluorochrome that binds to A-T-rich regions of DNA (Wallberg et al. 2016) and stains cells in blue. This makes it compatible with other fluorochromes that emit in both green and red channels. However, due to its semi-permeable properties, most studies have employed DAPI as a counterstaining for permeabilized cells (He et al. 2020; Zhang et al. 2020). Nevertheless, there is limited information in the literature regarding the use of DAPI as an indicator of cell viability in spermatozoa (Roy et al. 2015; Giaccagli et al. 2021; Gacem et al. 2023) and no studies have compared its performance with other fluorescent stains.

The aim of this work was to validate the use of DAPI stain as an alternative to the PI method for assessing sperm viability in various livestock species. This approach would free up the red channel in flow cytometry to use with other fluorochromes in multiparametric analysis.

## Materials and methods

### Semen collection

The experiments presented in the current work were performed using semen from three livestock species: goat, rabbit, and cattle.

Regarding goat spermatozoa, ejaculates were collected in different seasons throughout the year 2023, from 6 Murciano-Granadina breed adult bucks, housed in the Centro de Tecnología Animal, Instituto Valenciano de Investigaciones Agrarias (CITA-IVIA), in Segorbe, east Spain. The housing and care of animals, as well as the semen collection protocols for the male goats complied with European regulations for the care and use of animals for scientific purposes (Spanish Regulations RD 53/2013, modified by RD 118/2021). According to the legislation, semen collection with an artificial vagina is considered as a routine husbandry practice and, for this reason, does not require ethical approval. The detailed protocol for semen collection using artificial vagina was described by Silvestre et al. (2004). All samples were pooled and maintained cooled for 48 h, performing quality assays every 24 h. As to rabbit spermatozoa, the 41 males used for the study were housed at the Miguel Hernández University of Elche (Spain) farm. For male rabbits, the experimental procedures with animals were approved by the Directorate General of Agriculture, Livestock and Fisheries of the Generalitat Valenciana (2022/VSC/PEA/0226). Females were presented to the bucks to encourage ejaculation. Semen was collected using an artificial vagina prepared with hot water (45 °C) (Boiti et al. 2005; Serrano-Jara et al. 2025). Individual samples were diluted with Tris-acid citric and glucose and kept cooled at 4 °C for 24 h, until further analysis at University of Valencia. Concerning bull spermatozoa, commercial frozen semen doses from bovine artificial insemination center were used for the studies. For in vitro analysis, straws from 13 bulls were thawed in a water bath at 37 °C for 1 min and then diluted in PBS in a 1:1 ratio.

Concerning fresh samples, to increase the number of replicates and the range of the viability rate, they were kept refrigerated for several hours. FC analyses of caprine samples were performed at 0, 24 and 48 h of sperm storage, while rabbit samples were analyzed 24 h after their collection. In the case of the frozen-thawed semen, samples were kept at room temperature for two hours.

### Staining protocol and flow cytometry analysis

**Fig. 1:**
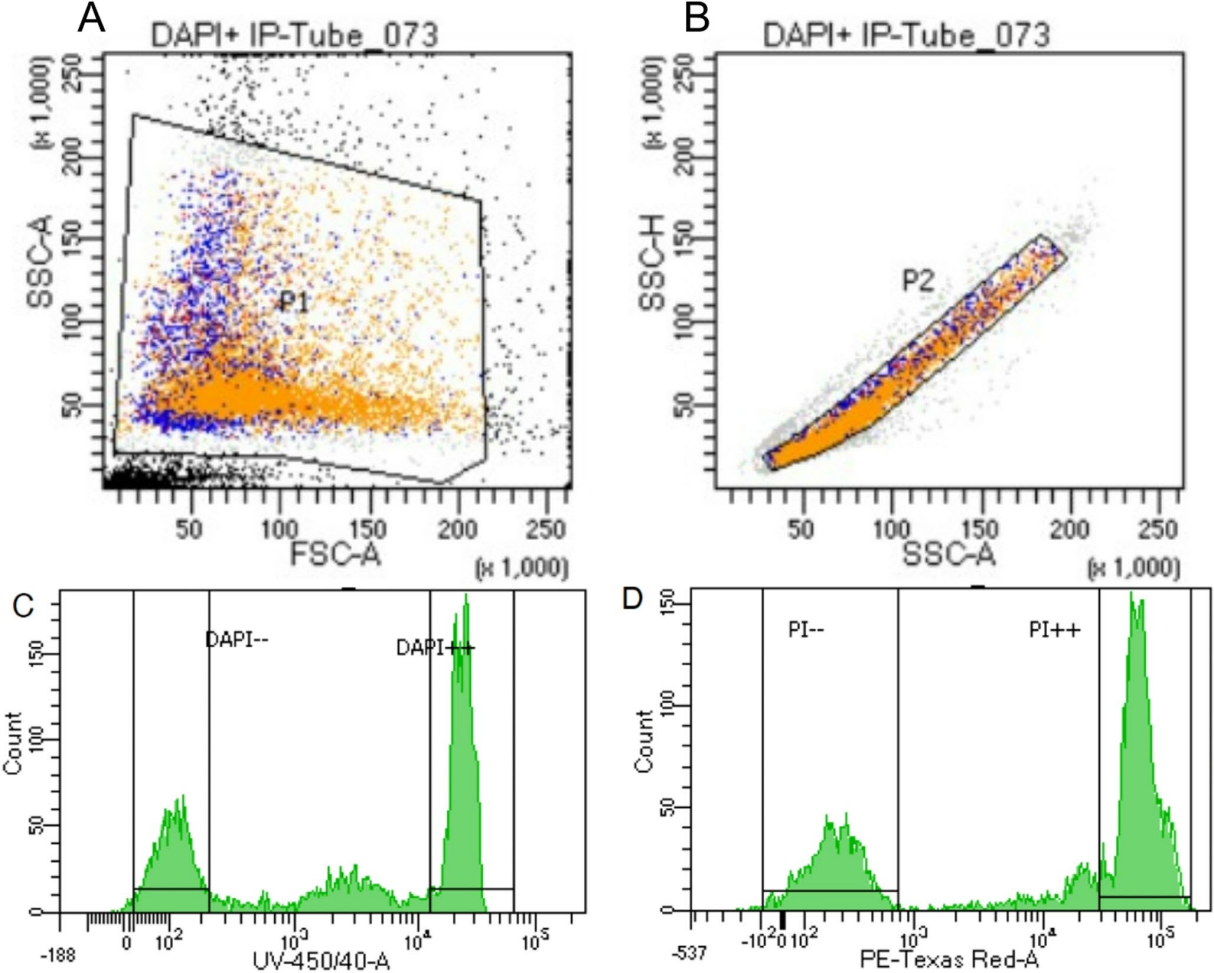
Cytometry gating strategy illustrated with bovine frozen semen. A: total events. P1 corresponds to the population selected once debris was sorted out; B: spermatozoa (P1). P2 corresponds to the population of single cells selected once doublets were sorted out; C: intensity histograms for DAPI; D: intensity histograms for PI

The concentration of the fluorochromes were adjusted by our laboratory based on optimization of the protocol. Aliquots of three µL of goat and rabbit fresh semen samples were stained with DAPI (D9542, Sigma-Aldrich) or PI (P4170, Sigma-Aldrich), and Dulbecco′s phosphate-buffered saline (PBS; D8537, Sigma-Aldrich) was added until the fluorochromes reached a final concentration of 7.5 µM PI or 2.9 µM DAPI. The same procedure was repeated for bull frozen-thawed samples, but the final concentrations of the fluorochromes were 11.2 µM PI and 1.4 µM DAPI. Samples stained with DAPI were incubated at 37 °C for 15 min, while PI samples were incubated for 5 min. Both DAPI and PI positive cells were considered as dead spermatozoa. FC assessments of unfixed samples were performed in the core facility of cell culture and flow cytometry at the Central Service for Experimental Research (SCSIE) of University of Valencia (Valencia). Samples were analyzed using a BD LSRFortessa flow cytometer (BD Biosciences, San Jose, CA, USA) configured with five lasers (355 nm UV, 405 nm violet, 488 nm blue, 561 nm yellow-green, and 640 nm red). The nominal output powers were approximately 15 mW for the laser used for DAPI (355 nm) and 50 mW for the laser used for PI (561 nm). Fluorescence emission was collected via multiple PMT detector arrays using a 450/40 nm bandpass for DAPI and a 610/20 nm bandpass for PI. Data were acquired with spatially separated lasers, a configuration that minimizes spectral spillover, reduces compensation requirements, and ensures reproducible multi-color panel performance. This cytometer was controlled using FACSDiva 8 software. Signals were amplified logarithmically, and photomultiplier settings were adjusted to each staining. A minimum of 10,000 cells per replicate was recorded. As we did not want to perform any counterstain, a gate was drawn on an FSC-A vs. SSC-A plot to exclude the debris (Fig. 1A), whereas single cells were discriminated using pulse-height vs. pulse-area for FSC parameter (Fig. 1B).

### Experimental design

**Fig. 2:**
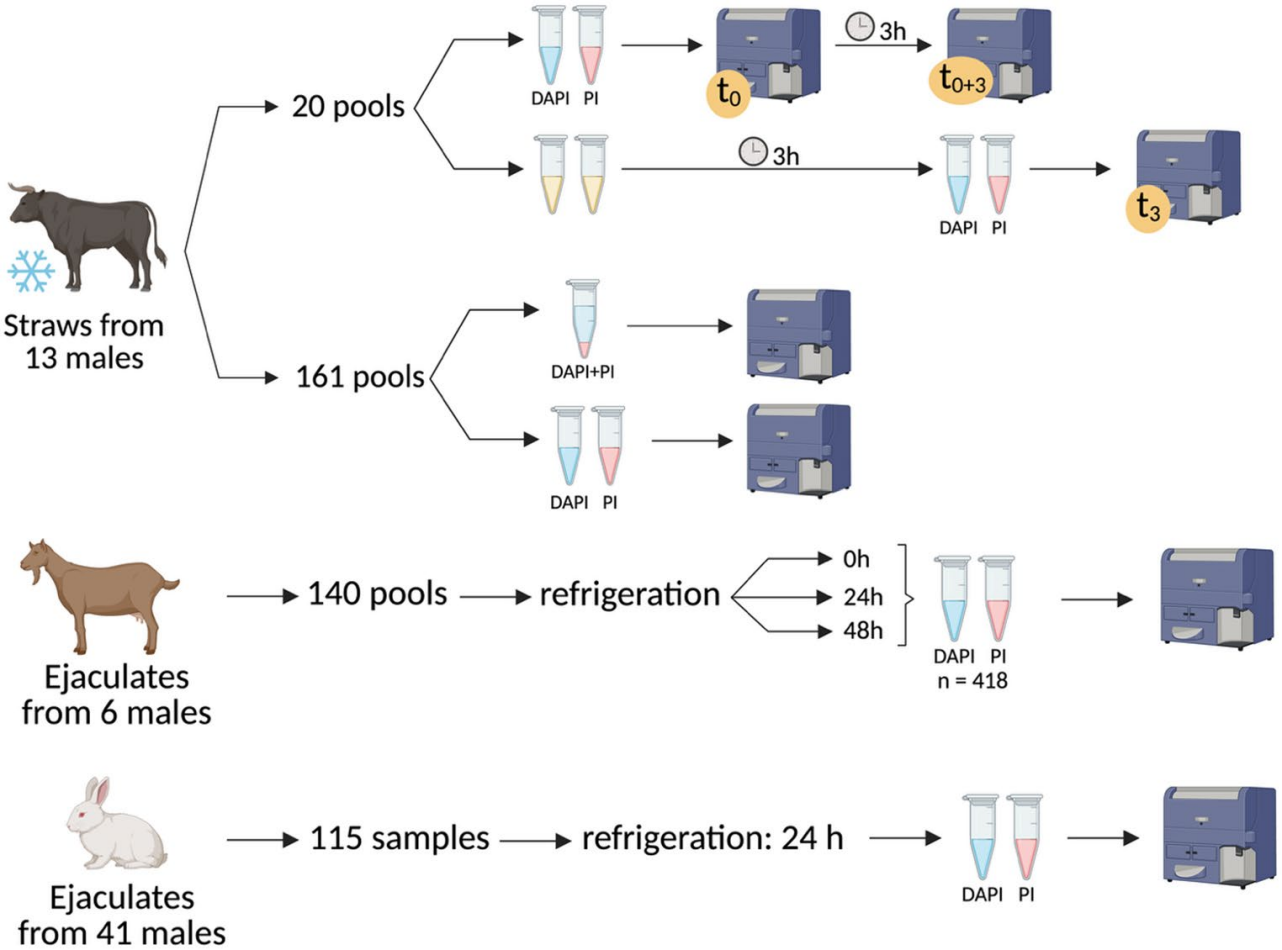
Graphical representation of the experimental design

To achieve the objective, three studies were performed (Fig. 2). In Studies 1 and 3, only bovine samples were used, while in Study 2 samples from the three species were assessed.

#### Study 1: Effect of incubation time with DAPI or PI on bovine sperm viability rate

DAPI might penetrate live cells, so we performed this study to determine the effect incubation time with or without fluorochromes had on the assessed viability rate of spermatozoa. Then, a total of 20 bovine semen samples were thawed and divided into three subsamples according to Table 1. Briefly, three groups were created depending on the time passed between the thawing of the frozen sample and the FC analysis (equilibration time). After thawing, the first subsamples (t0) were stained, and viability rate was assessed by FC immediately after the usual short incubation time. These same previously stained aliquots were conserved in the dark at room temperature for three additional hours and viability rate was analyzed again by FC (t0 + 3). The third subsamples (t3) were thawed and kept at room temperature for 3 h, afterwards they were stained, and viability rate was analyzed by FC. The same groups were repeated for every sample.

**Table 1:** Experimental design of study 1

Group	*Equilibration time (h)	**Incubation time
t_0_	0	5–15 min
t_0+3_	0	3 h
t_3_	3	5–15 min

**Incubation time: time between addition of the fluorochrome and the flow cytometry evaluation

*Equilibration time: time between sample thawing and addition of the fluorochrome

#### Study 2: Efficiency of DAPI as a viability stain on different livestock species

In this study, to generalize the use of DAPI as a viability stain, goat, rabbit, and bull sperm samples were stained with DAPI or PI and analyzed by FC. Each sample was split into two subsamples to compare the two different fluorescent stains DAPI and PI separately, following the staining protocol described above. For this assay, a total of 694 samples were assessed (418 goat samples, 115 rabbit samples, and 161 bull samples).

#### Study 3: Effect of intrasample variation of bovine semen samples on the correlation between fluorochromes

In this last study, to check for intrasample variation and a possible interaction between the two stains, 158 bovine semen samples were prepared with a mix of both DAPI and PI stains at the same time, following the same procedure.

### Statistical analyses

All analyses were performed using the SPSS software (IBM^®^ SPSS^®^ 28.0.1.1 for Windows; IBM corp., Armonk, NY, USA). The data normality was analyzed with the Kolmogorov-Smirnov test. Generalized linear models (GLM) with fixed factors were performed to assess the effects of different variables: in Study 1, the factors were incubation time and fluorochrome, and their double interaction; in Study 2: species and fluorochrome; for Study 3: protocol (together or separate) and fluorochrome. For each model, the significance of main effects and interactions was evaluated using a Wald Chi-Square test with a significance threshold of *p* ≤ 0.05. GLMs were performed under the default SPSS settings. In Studies 2 and 3, the viability rates obtained with both fluorochromes (DAPI or PI) were compared using a bivariate Pearson correlation, and the R^2^ value was calculated according to the linear fit for the scatter plot, and in Study 2 that correlation was also represented with a Bland-Altman plot to study the proportionality of the bias.

## Results

### Study 1: Effect of incubation time with DAPI or PI on bovine sperm viability rate

Results of the effect of incubation time with DAPI or PI on bovine sperm viability rate are shown in Table 2. No significant differences in the bovine sperm viability rate for both fluorochromes and three incubation times were found, ranging from 40.14 to 44.28%.

**Table 2:** Rate of viability of bovine thawed samples measured by the flow cytometer for both fluorochromes

Incubation time	Fluorochrome	Viability (%)
t_0_	PI	44.28 ± 1.92
	DAPI	40.76 ± 1.70
t_0+3_	PI	40.14 ± 4.53
	DAPI	39.39 ± 1.86
t_3_	PI	43.26 ± 1.64
	DAPI	42.79 ± 2.31

Incubation time: t_0_: semen samples were stained and assessed immediately after thawing; t_0+3_: t0 samples assessed after room temperature storage and in the dark for three additional hours; t_3_: after thawing, samples were kept at room temperature for 3&nbsp;h, afterwards they were stained and analyzed. PI: propidium iodide; DAPI: 4′,6-diamidino-2-phenylindole. Viability results are expressed in mean ± standard error

### Study 2: efficiency of DAPI as a viability stain on different livestock species

**Fig. 3:**
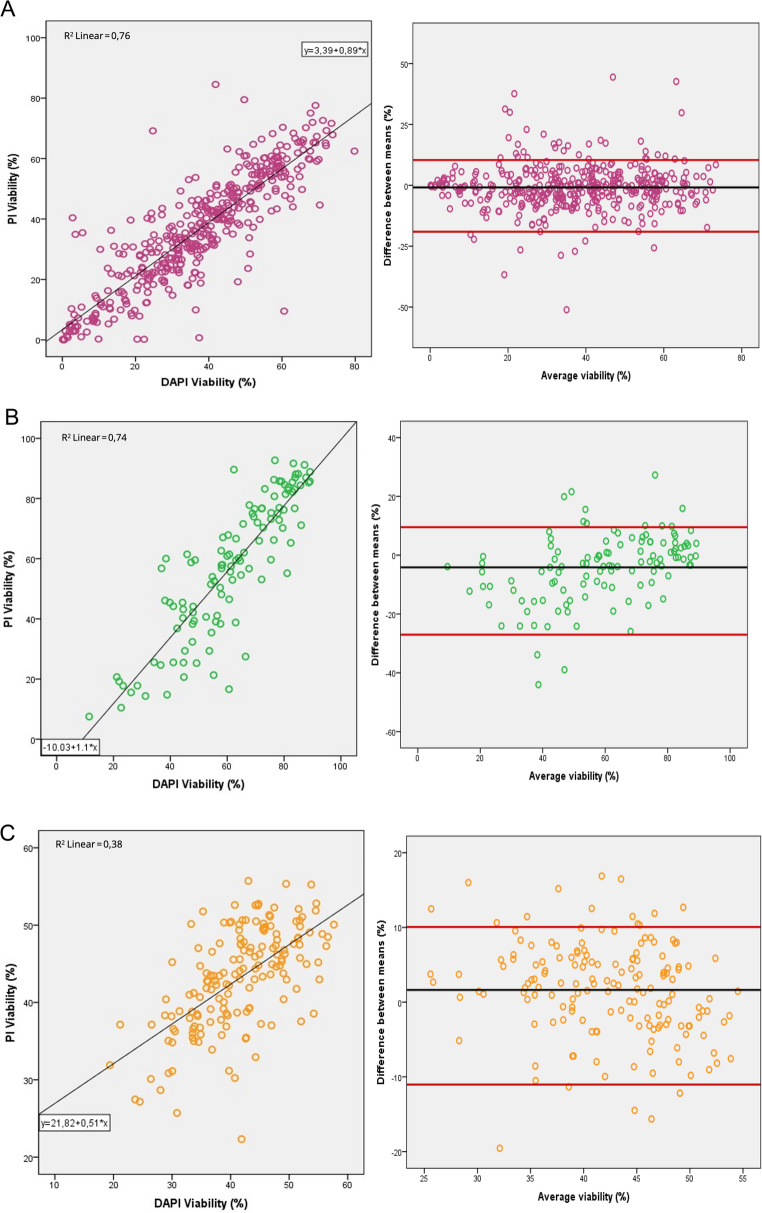
Dispersion plots for the sperm viability rate measured with PI and DAPI for each species. Left: linear fit for the dispersion. Right: Bland-Altman plot; y axis values correspond to the difference of the viability rate measured with PI minus the ones measured with DAPI; x axis values correspond to the mean of the viability rates measured with PI and DAPI; red lines: upper and lower values calculated for the 95% CI; black lines: mean of the difference. A: caprine fresh semen; B: rabbit fresh semen; C: bovine frozen-thawed semen. PI: fluorescent propidium iodide; DAPI: 4′,6-diamidino-2-phenylindole

Viability rates for each species are shown in Table 3, ranging from 36.65 to 37.50% in caprine samples, 57.26 to 61.39% in rabbit samples, and 43.10 to 41.47% in bovine samples (Table 3). Statistical analysis showed no differences between PI and DAPI in goat and rabbit semen and showed a significant correlation between stains of 0.87 (*P* < 0.01; R^2^ = 0.76) in caprine samples (Fig. 3A) and 0.86 (*P* < 0.01; R^2^ = 0.74) in rabbit samples (Fig. 3B). Nevertheless, both fluorochromes appeared to be significantly different in bovine samples, although they still presented a significant correlation between them of 0.61 (*P* < 0.01; R^2^ = 0.38) (Fig. 3C). In order to properly understand Fig. 3, a Bland-Altmann summary is shown at Table 4.

**Table 3:** Viability rate (mean ± standard error) measured by the flow cytometer for each stain and pearson correlation of the viability rate for each animal species

Species	Fluorochrome	Viability (%)	Correlation with DAPI rate
Caprine	PI	36.65 ± 0.90	0.87*
	DAPI	37.50 ± 0.89	1
Rabbit	PI	57.26 ± 2.13	0.86*
	DAPI	61.39 ± 1.68	1
Bovine	PI	43.10 ± 0.52 a	0.61*
	DAPI	41.47 ± 0.62 b	1

* Correlation is significant at the 0.01 level (2-tailed) according to Pearson correlation. DAPI: 4′,6-diamidino-2-phenylindole; PI: propidium iodide. Different lowercase letters (a, b) indicate the significant differences between fluorochromes (*P* < 0.05) for the same species. Caprine and rabbit results correspond to fresh samples, whereas bovine results correspond to frozen-thawed samples

**Table 4:** Summary of Bland–Altman analysis by species. The mean bias corresponds to the average difference between fluorochromes, and the lower and upper limits of agreement were calculated as bias ± 1.96 × SD of the differences. Values are reported with their 95% confidence intervals

Species	Bias (95% CI)	Upper LoA (95% CI)	Lower LoA (95% CI)
Goat	−0.85	10.39	−19.04
Rabbit	−4.13	9.51	−27.03
Bull	1.64	10.06	−11.03

**Fig. 4:**
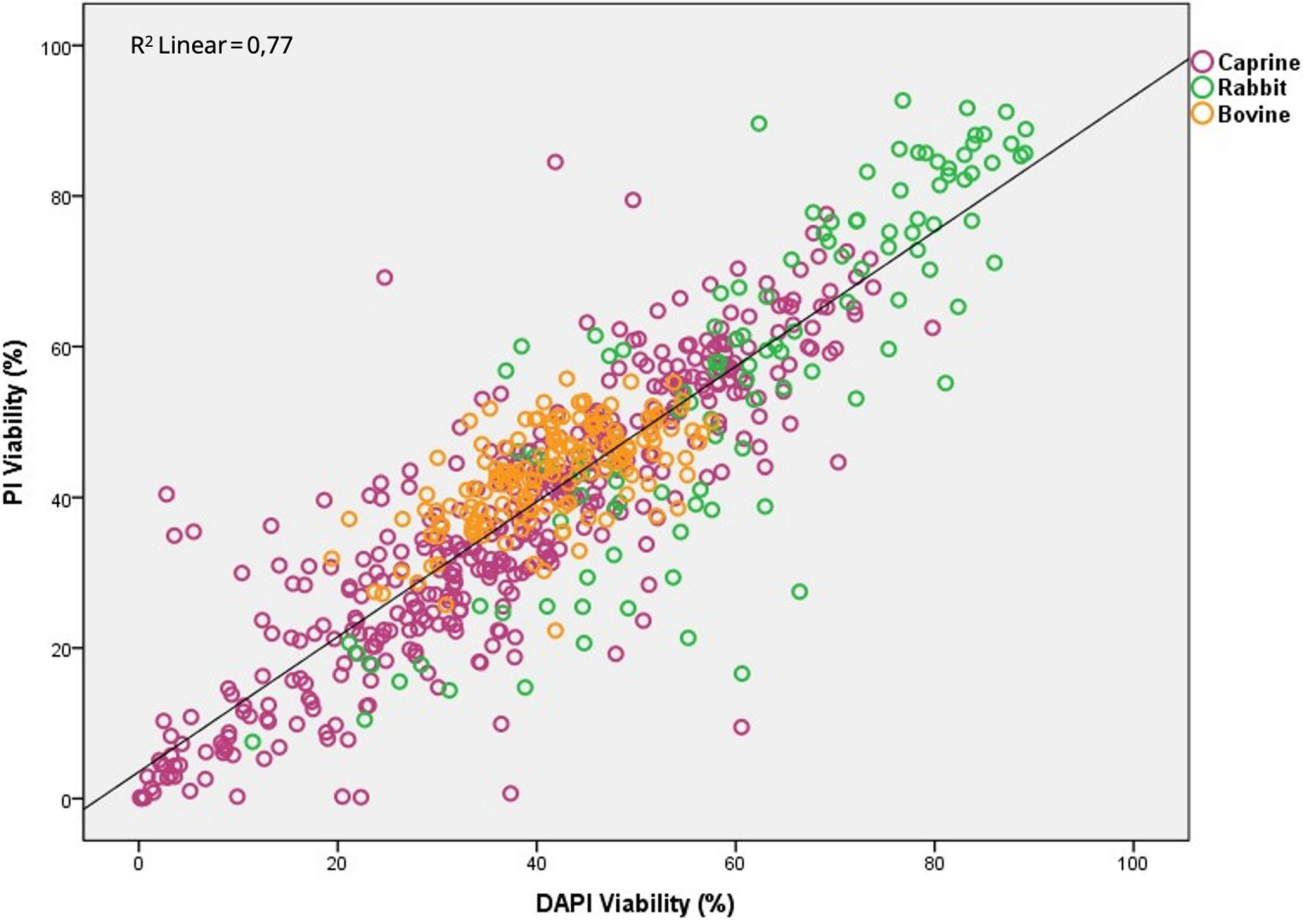
Dispersion plot for the viability rate measured with PI and DAPI. Different colors represent the species. Linear fit was performed. PI: propidium iodide; DAPI: 4′,6-diamidino-2-phenylindole. Caprine and rabbit results correspond to fresh samples, whereas bovine results correspond to frozen-thawed samples

As a summary, viability rate results for all three species together are shown in Table 5. Values were not different and ranged from 41.56 to 42.38% for PI and DAPI, respectively. When analyzing bivariate Pearson correlation, a significant value of 0.88 was observed (*P* < 0.01; Table 5), with an R^2^ value of 0.77 (Fig. 4).

**Table 5:** Percentage of viability rate (mean ± standard error) measured by the flow cytometer for each stain and pearson correlation of the viability rate regardless of the species

Fluorochrome	Viability (%)	Correlation with DAPI rate
PI	41.56 ± 0.72	0.88**
DAPI	42.38 ± 0.70	1

**Correlation is significant at the 0.01 level (2-tailed) according to Pearson correlation. PI: propidium iodide; DAPI: 4′,6-diamidino-2-phenylindole

### Study 3: effect of intrasample variation of bovine semen samples on the correlation between fluorochromes

**Fig. 5:**
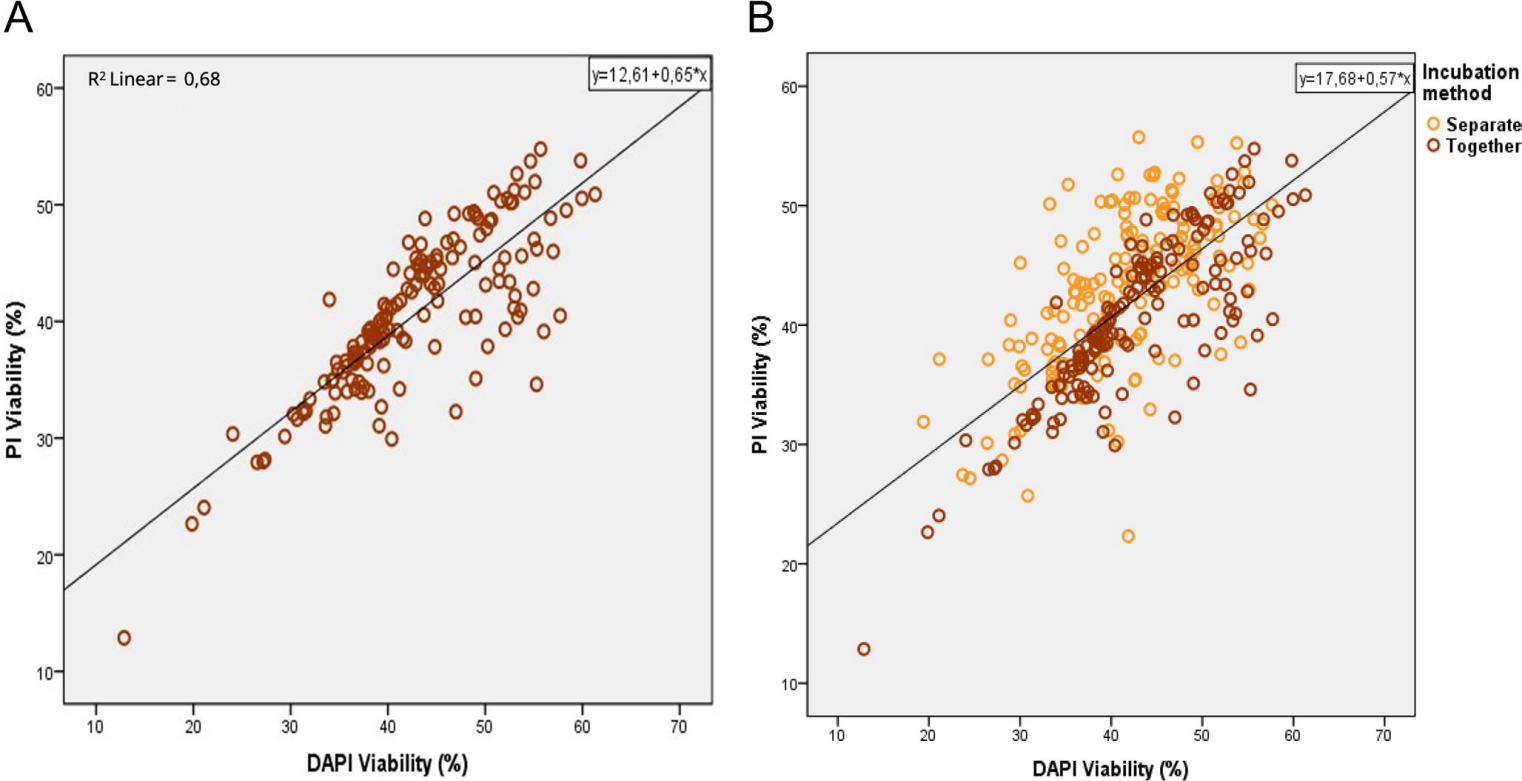
Dispersion plot for the sperm viability rate between PI and DAPI in Study 3 aimed to analyze intrasample variation in fozen-thawed bovine samples. A: Dispersion plot for the viability rate measured when PI and DAPI were incubated together in the same subsample (Study 3). B: Superposition of results obtained in Study 3 (brown) with results from Study 2 (orange), where PI and DAPI were separated into different subsamples. PI: propidium iodide; DAPI: 4′,6-diamidino-2-phenylindole

The viability rate (Table 6) for bovine semen samples measured with PI or DAPI after both fluorochromes were placed together presented a correlation of 0.82 (*P* < 0.01; R^2^ = 0.68) between both fluorochromes (Fig. 5A), yet the average PI viability rate (40.53%) appeared to be statistically significant and different to the one measured with DAPI (42.68%). However, these differences were not observed when the totality of bull samples (the ones from Study 2 and Study 3) were analyzed together, showing viability rate values of 41.83 and 42.07 for PI and DAPI, respectively (*P* > 0.05; Table 7).

**Table 6:** Comparison of the viability rate between PI and DAPI for bovine samples when PI and DAPI were incubated together. Left) percentage of live cells measured by the cytometer for each stain. Right) pearson correlation

Fluorochrome	Viability (%)	Correlation with DAPI rate
PI	40.53 ± 0.54 b	0.82 ^*^
DAPI	42.68 ± 0.68 a	1

*Correlation is significant at the 0.01 level (2-tailed) according to Pearson correlation. PI: propidium iodide; DAPI: 4′,6-diamidino-2-phenylindole. Viability results are expressed in mean ± standard error, different lowercase letters (a, b) indicate the significant differences between fluorochromes (*P* < 0.05)

**Table 7:** Comparison of the viability rate between PI and DAPI for every bovine sample regardless of the incubation protocol of the fluorochrome. Left: percentage of live cells measured by the cytometer for each stain. Right: pearson correlation

Fluorochrome	Viability (%)	Correlation with DAPI rate
PI	41.83 ± 0.38	0.69^**^
DAPI	42.07 ± 0.46	1

**Correlation is significant at the 0.01 level (2-tailed) according to Pearson correlation. PI: propidium iodide; DAPI: 4′,6-diamidino-2-phenylindole

**Fig. 6:**
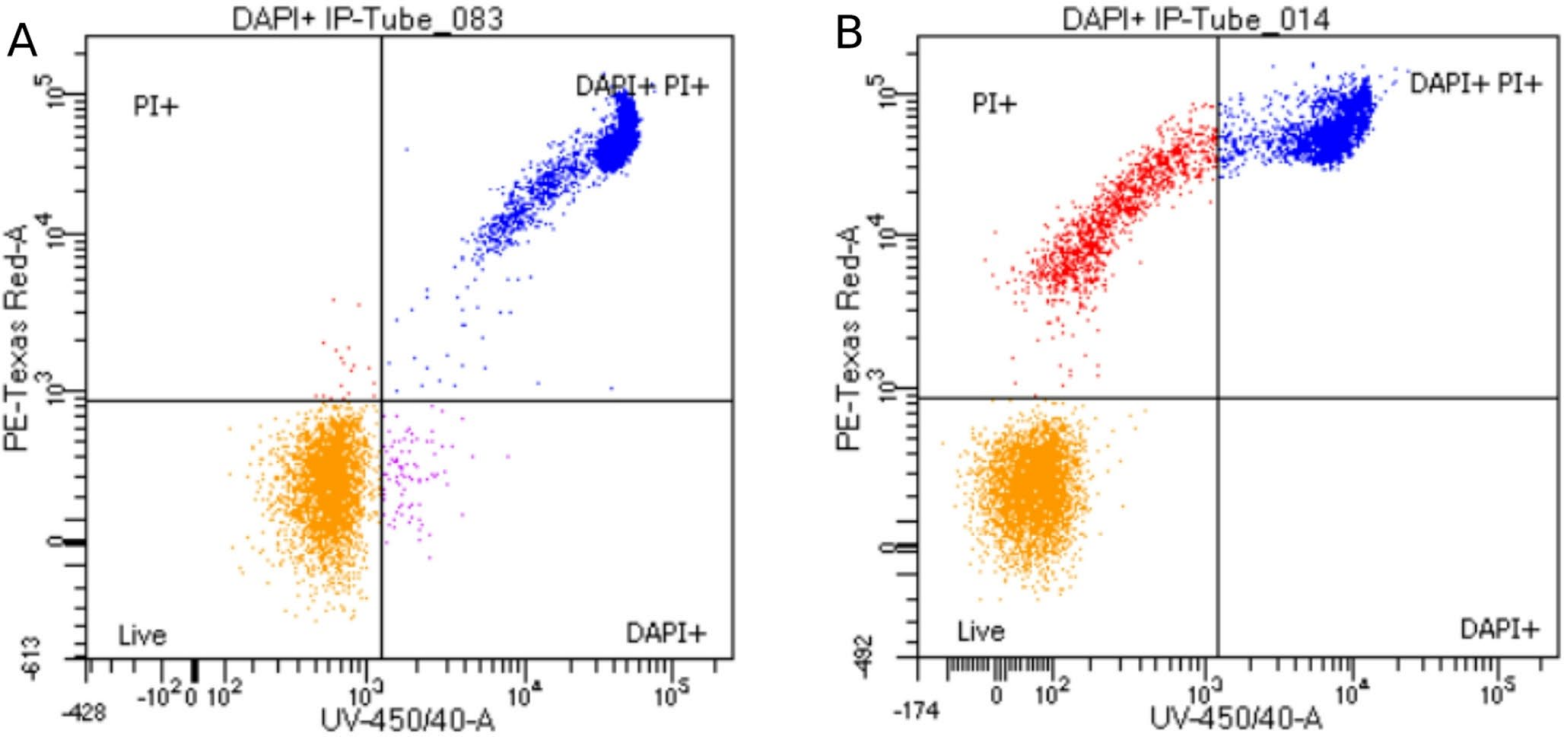
Different flow cytometry outputs after bovine thawed spermatozoa were stained with both fluorochromes simultaneously. A: Overlap between DAPI+ and PI+ cells. The lower left quadrant (orange population) corresponds to spermatozoa not stained with either fluorochromes (live cells; PI-/DAPI-); meanwhile the upper right quadrant (blue population) corresponds to dead cells marked with both fluorochromes. B: In some cases, a different population in the upper left quadrant that was marked PI+ but DAPI- was observed (red). PI: propidium iodide; DAPI: 4′,6-diamidino-2-phenylindole

Also, the FC output showed that cells marked with PI were also stained with DAPI, allowing us to differentiate between two populations: dead (PI+/DAPI+) and alive (PI-/DAPI-) sperm cells (Fig. 6 A). Nevertheless, in approximately 30% of the samples, we found a third cell population that appeared to be PI+/DAPI- (Fig. 6B).

## Discussion

The plasma membrane is the structure that separates the spermatozoa from the external environment, and its integrity is crucial for maintaining physiological homeostasis. It enables the selective transport of substances and protects the sperm from extracellular damage (Peña et al. 2018). It also plays a key role in sperm capacitation, undergoing structural and molecular remodeling that subsequently leads to sperm hyperactivation (Parrish 2014). Moreover, the plasma membrane is involved in gamete recognition, particularly in chemotaxis processes and the acrosomal reaction (Sutovsky 2018). The viability rate has also been shown to correlate with other sperm quality parameters such as acrosome integrity, motility, or DNA fragmentation (Samplaski et al. 2015; Yániz et al. 2017). As we observed in ovine sperm (Santolaria et al. 2015), several studies showed a positive correlation between membrane integrity and fertility (Oliveira et al. 2013; Sellem et al. 2015; Ahmed et al. 2017), highlighting the importance of accurate measurement and evaluation. In this context, some authors have reported results where motility rates exceed viability rates (Oliveira et al. 2013; Sellem et al. 2015), underscoring the significance of the viability assessment protocol. However, discrepancies also exist, as some studies refute correlation between in vitro fertility and membrane integrity (Brito et al. 2003).

Thus, identifying the appropriate combination of fluorochromes to evaluate the viability and other sperm parameters is essential. Previous studies have demonstrated that DAPI can be used to evaluate both apoptotic and necrotic cells (Atale et al. 2014), unlike other fluorochromes, such as Annexin V which specifically detects apoptosis(Hoogendijk et al. 2009) but is unsuitable for necrosis evaluation unless combined with other vital stain. The significance of different death processes when selecting fluorochromes was already highlighted by Huang et al. (2024), who showed that DAPI overestimated the viability rates of Low-Temperature/Nutrient-Deprived cells but not on Heat-Killed cells, thus suggesting a possible action mechanism that depends on the type of cell death (Huang et al. 2024). Moreover, limiting the evaluation of semen quality samples to sperm viability alone is insufficient. Estimating spermatozoa fertility potential is inherently complex and requires simultaneous analysis of multiple parameters. For instance, Sellem et al. (2015) proved that increasing the number of parameters studied when evaluating semen quality improved the predictability of field fertility (Sellem et al. 2015). Among the parameters that can be assessed alongside viability is mitochondrial function (Peña et al. 2018). In this context, DAPI is particularly advantageous for mitochondrial function analysis, as most studies utilize the red-orange channel for mitochondrial staining (Treulen et al. 2018; Zhang et al. 2019; Thananurak et al. 2020; Varela et al. 2020), blocking it for the PI labelling.

Additionally, DAPI offers a cost-effective and convenient alternative, as it does not require a UV for detection and can also be excited by a blue laser. Moreover, it exhibits greater photostability than other blue stains, such as Hoechst, making it less susceptible to long-term degradation (Chazotte 2011). This will facilitate its use not only with fresh samples but also with fixed preparations, enabling labelled cells to be preserved until further analysis without losing their fluorescence (Peña et al. 2018). Furthermore, while DAPI may have a low-level mutagenic effect, PI is a known mutagen with higher toxicity, which complicates its use and disposal (Atale et al. 2014).

Few studies have employed DAPI to assess sperm membrane integrity, with concentrations ranging from 0.05 to 2.9 µM (Roy et al. 2015; Giaccagli et al. 2021; Gacem et al. 2023). Nevertheless, DAPI has primarily been used in permeabilized or dead cells (He et al. 2020; Zhang et al. 2020). This can be due to DAPI being perceived as a permeable stain that is combined with PI to assess cell viability (Jenkins et al. 1997; Kainulainen et al. 2002; Johnson and Criss 2013), so that some viable cells could be stained after long incubation times. However, results from Study 1 showed no significant differences in viability rates measured with DAPI when incubation time was increased up to 3 h.

An extensive body of literature is available regarding the use of PI as a vital stain and the viability rates obtained in our study were consistent with previous findings in goat (Caamaño et al. 2023), rabbit (Kulíková et al. 2017), and bull spermatozoa (Brito et al. 2003). Nevertheless, discrepancies were observed concerning the concentration of fluorochrome used. Most studies reported PI concentrations for FC ranging from 0.06 to 3 µM (Kulíková et al. 2017; Peña et al. 2018; Caamaño et al. 2023). However, some studies employed higher PI concentrations (up to 160 µM) and still obtained results consistent with our data (Mendoza et al. 2021). According to Williams et al. (1998), who tested PI concentrations ranging from 1.5 to 59.8 µM, the optimal concentration of PI was 7.5 µM (Williams et al. 1998). In our assays, PI concentrations between 7.5 and 11.2 µM were used. Nonetheless, considering that Willliams’ protocols were developed for fluorescence microscopy, reducing the amount of fluorochrome might be advisable when performing analyses with FC due to it is high sensitivity.

Concerning the use of DAPI to assess viability rates across different animal species, the results revealed significant interspecific differences in viability rates. These variations align with existing literature, which has documented variability in seminal parameters not only across species (Van der Horst 2021) but also among breeds (Wysokińska 2022). Conversely, no differences were observed between PI and DAPI in goat and rabbit samples, where the viability rates obtained with both fluorochromes were highly correlated, validating using DAPI as a substitute for PI.

In bull semen, however, while the performance of the two fluorochromes was still correlated, significant differences in viability rates were identified between PI and DAPI. A biological explanation for this could be that bovine spermatozoa are known to present highly compacted chromatin (Ribas-Maynou et al. 2021), which could potentially affect the accessibility of nuclear fluorochromes. This effect would be particularly relevant for DAPI, as it binds preferentially to AT-rich regions of DNA (Ligasová and Koberna 2021) and may encounter steric hindrance under higher degrees of chromatin condensation. Such a mechanism could partly explain if higher viability rates were observed with DAPI (indicating reduced binding of the fluorochrome), but in Study 2 the opposite trend was found, with a higher percentage of DAPI-stained cells compared to PI. This finding prompted the design of Study 3 to determine whether these differences were attributable to intrasample variation, but the results of the study continued to demonstrate differences between fluorochromes. However, by pooling all data, the increased sample size eliminated the differences between fluorochromes. This conclusion is further supported by the fact that, despite significant differences in mean values within the smaller sample population, the correlation between PI and DAPI was still high, reaching up to 82,51% when both stains were incubated together.

In this study, we found an unexpected discrepancy in viability rates in a cell population positive for PI but not for DAPI. Given that DAPI is more permeable than PI, we would have anticipated the opposite result (DAPI+/PI-), but that did not happen. Huang and collaborators (2024) also described a higher proportion of viable Jurkat cells measured with DAPI than with PI, but they attributed this discrepancy to differences in staining time, suggesting that certain dead cells might remain unstained with DAPI after 30 min of incubation, despite being already marked with PI (Huang et al. 2024). These findings contrast with the results of Study 1, where no differences in cell labelling were observed after 3 h of incubation. Huang et al. (2024) also hypothesized that variations in fluorescence intensity might arise from PI’s larger molecular size, resulting in a stronger fluorescent signal. Although this explanation could be relevant in fluorescence microscopy, it is unlikely to apply in FC. A straightforward approach to address this issue would involve analyzing fluorescence intensity units instead of dichotomously classifying cells as positive or negative for a given stain. Such an approach may provide a more precise evaluation of the membrane integrity.

Moreover, the mechanisms by which both fluorochromes interact with DNA are slightly different: PI is a base intercalator and squeezes in between the base pairs, interacting with DNA and RNA both within and outside the nucleus, without a specific nucleotide preference; while DAPI needs to specifically intercalate with the minor groove of the DNA (Ligasová and Koberna 2021). The ability of PI to bind extracellular double-stranded nucleic acids may result in a higher emission signal in the red channel, potentially being misinterpreted as an increase in cell mortality. Such binding has been observed in both extracellular (Rosenberg et al. 2019) and cytosolic DNA (Rieger et al. 2010). A potential explanation for this phenomenon lies in PI’s non-specific binding to DNA, that allows it to interact with extranuclear genetic material, such as cytosolic RNA or exogenous DNA fragments released during cell death. However, sperm cells have a very reduced cytoplasm (Cooper 2005), making the likelihood of extranuclear labeling with PI relatively low. This should not be a problem with DAPI since it specifically binds to the minor groove of DNA helix (Ligasová and Koberna 2021), thus minimizing its interaction with external nucleic acids. Additionally, the highly compacted nature of sperm chromatin (Ward 2010) may hinder DAPI molecules from accessing the minor groove, potentially leaving some dead cells unstained.

Alternatively, a plausible explanation for the observed differences could involve changes in the permeability of the plasma membrane to PI. Previous studies have demonstrated that PI can enter live cells with elevated membrane potential (Kirchhoff and Cypionka 2017). PI has two positive charges on its structure (Ligasová and Koberna 2021), one of these charges is primarily responsible of its non-permeable nature as it remains free to interact with the surroundings. An increase on the membrane potential could amplify the ion-motive force for cations, which facilitates PI transport into the cell (Kirchhoff and Cypionka 2017). DAPI could be a way of avoiding this since it is a neutral molecule and no charges are free to interact with the membrane (Ligasová and Koberna 2021). However, altered membrane potential is not the sole factor affecting PI permeability. Changes have also been observed following membrane repair after heat shock (Davey and Hexley 2011) and through pannexin channels that allow the flow of PI through the membrane of viable spermatozoa in stallion, ram (Savvulidi et al. 2023), and dog semen (Torres et al. 2017).

Another potential explanation for the observed differences could be the proportion of dead cells in the sample. However, there are some disagreements in this regard. Huang et al. (2024) reported greater differences in viability measurements when the sample exhibited a low overall viability rate, whereas (Chan et al. 2012) observed more pronounced differences at higher viability rates. Both studies were performed with Jurkat cells, suggesting that variability in cell type might not be the only explanation for these discrepancies. Nevertheless, our Bland-Altman results showed no trend in this aspect, with differences distributed across samples with both low and high viability rates. Furthermore, our correlation data confirms the equivalent performance of PI and DAPI in assessing cell viability.

## Conclusions

The present study validates the use of DAPI as an alternative to the PI method for assessing sperm viability in livestock species, demonstrating that using DAPI as a vital stain instead of PI can free the red fluorescence channel for additional analysis without compromising the accuracy of the viability measurements, in our studied conditions.

## Data Availability

The data that support the findings of this study are available from the corresponding author upon reasonable request.
